# Evaluating the Impact on Clinician Diagnostic Confidence and Management Following Disclosure of Alzheimer's Disease Probability Informed by Plasma pTau181 Levels in Memory and Cognition Clinic Patients: A Before‐and‐After Study

**DOI:** 10.1002/alz70856_099163

**Published:** 2025-12-25

**Authors:** Johannes C. Michaelian, Azadeh Feizpour, James C. Vickers, Jane E. Alty, Jessica M. Collins, Vincent Dore, Catriona Ireland, Anna E. King, Michael Woodward, Sharon L. Naismith, Christopher C. Rowe

**Affiliations:** ^1^ Healthy Brain Ageing Program, Brain and Mind Centre, School of Psychology, Faculty of Science, University of Sydney, Camperdown, NSW, Australia; ^2^ Charles Perkins Centre, University of Sydney, Camperdown, NSW, Australia; ^3^ Department of Molecular Imaging & Therapy, Austin Health, Melbourne, VIC, Australia; ^4^ Wicking Dementia Research and Education Centre, University of Tasmania, Hobart, TAS, Australia; ^5^ The Australian e‐Health Research Centre, Commonwealth Scientific and Industrial Research Organisation, Melbourne, VIC, Australia; ^6^ Department of Continuing Care, Austin Health, The University of Melbourne, Melbourne, VIC, Australia; ^7^ Healthy Brain Ageing Program, Brain and Mind Centre, School of Psychology, Faculty of Science, University of Sydney, Australia, Camperdown, NSW, Australia

## Abstract

**Background:**

Recent global breakthroughs have enabled blood‐tests to detect key proteins implicated in the pathophysiology of Alzheimer's disease (AD). Clinical implementation of a non‐invasive, cost‐effective blood‐test could improve diagnostic accuracy, particularly in the early stages of the disease, facilitating timely commencement of disease‐modifying therapies. In this study, we evaluated the impact of disclosing a patient's AD probability, determined by plasma pTau181 levels, on clinicians' diagnostic confidence and management.

**Method:**

We conducted a before‐and‐after study in three Australian Memory and Cognition Clinics, enrolling routine patients aged ≥50 years and MMSE scores ≥23. Blood was collected at initial assessment, followed by a multidisciplinary evaluation to determine diagnosis, management, and clinician confidence prior to receiving blood‐test results. Plasma pTau181 levels were measured on the Quanterix Simoa™ platform; and results were reported to clinicians as probably negative, indeterminate or probably positive, based on two thresholds set at 90% sensitivity and 90% specificity for amyloid‐beta PET positivity. The impact of the blood‐test result on the initial diagnosis, management and confidence was documented after clinicians reviewed the report.

**Result:**

A total of 113 participants (mean age: 73.6±7.8; mean MMSE: 27.7±2.5) with dementia (*n* = 17, 15.0%), mild cognitive impairment (*n* = 48, 42.5%) and subjective cognitive decline (*n* = 48, 42.5%) were enrolled. Mean diagnostic confidence following initial clinical assessment was moderate‐to‐high (77.2%). Most blood‐test results received were probably negative (*n* = 81, 71.7%), followed by indeterminate (*n* = 24, 21.2%) and probably positive (*n* = 8, 7.1%). In 14 cases (12.4%), blood‐test results (probably negative: *n* = 8; probably positive: *n* = 3; indeterminate: *n* = 3) changed clinician diagnosis, increasing mean diagnostic confidence in these patients from low‐to‐moderate (63.1%) to moderate‐to‐high (78.6%), with a mean increase of 15.4%. Two‐thirds of clinicians would recommend the clinical use of such blood‐tests with further refinement.

**Conclusion:**

This study highlights the potential of a blood test for AD in clinical practice, enhancing diagnostic accuracy in cases with low‐to‐moderate diagnostic confidence.

